# Metformin modifies hormone changes associated with androgen deprivation therapy for prostate cancer

**DOI:** 10.1530/EO-25-0114

**Published:** 2026-07-01

**Authors:** Mathew Gorman, Nawaid Usmani, Michael N Pollak, Sunita Ghosh, Arun Elangovan, Julian Kim, John Thoms, Myriam Bouchard, Michael Peacock, Ye Wang, Neil E Fleshner, Holly Campbell, Eric Vigneault, Francois Vincent, Alan So, Fabio L Cury, Harvey C Quon, Ryan G Carlson, Carole Lambert, Laurence Klotz, Kim Chi, Michael Brundage, Kerry S Courneya, Bernhard J Eigl

**Affiliations:** ^1^ University of Alberta, Edmonton, Alberta, Canada; ^2^ Departments of Medicine and Oncology, McGill University, Montreal, Quebec, Canada; ^3^ Department of Public Health Sciences, Henry Ford Hospital, Detroit, Michigan, USA; ^4^ Saskatoon Cancer Centre, Saskatoon, Saskatchewan, Canada; ^5^ Department of Radiation Oncology, CancerCare Manitoba, Winnipeg, Manitoba, Canada; ^6^ Dr H. Bliss Murphy Cancer Centre, St. John’s, Newfoundland and Labrador, Canada; ^7^ Centre Intégré Universitaire de Santé et de Services Sociaux de L’ Estrie-Centre Hospitalier Universitaire de Sherbrooke, Sherbrooke, Quebec, Canada; ^8^ BC Cancer – Vancouver Center, Vancouver, British Columbia, Canada; ^9^ University of British Columbia, Vancouver, British Columbia, Canada; ^10^ Lady Davis Institute for Medical Research, Jewish General Hospital and McGill University, Montreal, Quebec, Canada; ^11^ University of Toronto, Toronto, Ontario, Canada; ^12^ Dalhousie University, Halifax, Nova Scotia, Canada; ^13^ Centre de Recherche CHU de Québec Université Laval, Québec City, Quebec, Canada; ^14^ Centre intégré universitaire de santé et de services sociaux de la Mauricie-et-du-Centre-du-Québec, Trois-Rivières, Quebec, Canada; ^15^ McGill University Health Centre, Montreal, Quebec, Canada; ^16^ Division of Radiation Oncology, Arthur J.E. Child Comprehensive Cancer Centre, University of Calgary, Calgary, Alberta, Canada; ^17^ Shirley and Jim Fielding Northeast Cancer Centre, Sudbury, Ontario, Canada; ^18^ Centre hospitalier de l’Université de Montréal, Montreal, Quebec, Canada; ^19^ Sunnybrook Health Sciences Centre, Toronto, Ontario, Canada; ^20^ Kingston Regional Cancer Centre, Kingston, Ontario, Canada

**Keywords:** metformin, prostate cancer, androgen deprivation therapy, ADT, hormone changes, PRIME

## Abstract

Androgen deprivation therapy (ADT) is regularly used to treat locally advanced and metastatic prostate cancer (PCa) with toxicities, including metabolic syndrome (MS) and associated adverse hormonal changes. This study evaluated whether metformin mitigates changes in laboratory biomarkers associated with MS and type II diabetes mellitus (T2DM) in PCa patients receiving ADT. PRIME is a phase III double-blind, randomized controlled trial in which 166 normoglycemic patients with PCa receiving ADT were randomized to receive metformin or placebo. For this study, 47 patients from the metformin arm and 32 patients from the placebo arm underwent serum collection and analysis of the following analytes at baseline, 9, and 12 months: IGF-1, IGFBP1, IGFBP2, IGFBP3, IGFBP7, leptin, adiponectin, GDF15, insulin, C-peptide, GIP, GLP-1, and IL-6. Independent *t*-tests were used to determine whether significant changes in analytes were evident in patients receiving metformin vs placebo and to evaluate analyte changes from baseline for the metformin and placebo groups separately. Mean leptin values increased markedly in the placebo group and significantly less in the metformin group across all time points. Significant improvements were also observed in IGFBP1, IL-6, C-peptide, and GLP-1 with metformin compared with placebo. GDF15 and IGFBP3 significantly increased compared with baseline with ADT alone. This study demonstrates that metformin can mitigate biomarker changes induced by ADT associated with an increased risk of T2DM and MS. In addition, the attenuated increase in leptin signals a potential for improved PCa outcomes, as high leptin values have been correlated with aggressive disease and worse prognosis.

## Introduction

Androgen deprivation therapy (ADT) is a pivotal treatment component in patients diagnosed with locally advanced and metastatic prostate cancer (PCa) (1, 2). Luteinizing hormone-releasing hormone agonists and antagonists are highly effective forms of ADT, which act by inhibiting testosterone production, thus improving disease-free survival, local control, metastasis-free survival, progression-free survival, and overall survival outcomes (3, 4). However, ADT is associated with an increased risk of insulin resistance and hyperglycemia (5). This can lead to metabolic syndrome (MS) and resultant complications, including weight gain, type II diabetes mellitus (T2DM), hypertension, and cardiovascular diseases (1, 6, 7, 8).

Metformin has demonstrated effectiveness in the management and prevention of T2DM and remains as the widely recommended first-line therapy in patients with newly diagnosed T2DM (9, 10). It has been shown to improve glycemic control, cardiovascular outcomes, weight gain, and overall survival (11, 12, 13). In the PRIME trial, it was hypothesized that the addition of metformin to ADT in PCa patients would result in a decreased incidence of MS. The results of this trial showed the prevalence of MS was not significantly different between the metformin and placebo groups. However, multiple secondary endpoints, including body weight, waist circumference, BMI, and HbA1c, were all significantly improved with metformin compared with placebo (14). As a part of the PRIME trial, serum samples were collected to study laboratory analytes shown to be associated with MS and/or T2DM. These analytes included IGF-1 (15, 16, 17), IGFBP1 (16, 17), IGFBP2 (16), IGFBP3 (15, 16), IGFBP7 (18), leptin (19, 20), adiponectin (21), GDF15 (22), insulin (23, 24), C-peptide (25, 26), GLP-1 (27), GIP (27, 28), and IL-6 (29, 30,31). The clinical impact of adverse changes in these metabolites on T2DM and MS has been evaluated only in cross-sectional studies, case–control studies, and population-based analyses (15, 16, 17, 18, 19, 20, 21, 22, 23, 24, 25, 26, 27, 28, 29, 30). Rigorous studies in the context of androgen deprivation for prostate cancer treatment are lacking.

We hypothesized that PCa patients receiving ADT would have unfavorable changes in laboratory biomarkers associated with MS and T2DM and that metformin would mitigate these changes.

## Materials and methods

### PRIME study overview

The PRIME study was a phase III multicenter, double-blind, randomized controlled trial conducted across 17 Canadian institutions. Approval was obtained from the research ethics boards of all participating institutions prior to initiation, and the trial is registered with ClinicalTrials.gov (NCT03031821).

The primary objective of the study was to determine whether the addition of metformin to ADT decreased the prevalence of MS at 18 months when compared to placebo. The study included participants diagnosed with localized, metastatic, or biochemically recurrent PCa and received ADT with either luteinizing hormone-releasing hormone agonists or antagonists for at least 9 months. Using a web-based data system (REDCap), participants were randomly assigned 2:1 to receive either metformin 850 mg or placebo orally twice daily for a total duration of 18 months. Interventions were started within 7 business days of randomization, and double-blinding with identical placebo controls was employed. Anthropomorphic and biochemical assessments were taken at 9, 12, 18, and 24 months to compare the overall prevalence of MS between the two groups.

### Blood sample collection

Among the patients who participated in the PRIME study, 47 from the metformin arm and 32 from the placebo arm volunteered to provide serum samples for correlative analysis. Fasting and postprandial serum samples were collected at baseline, 9, and 12 months to assess the following analytes: IGF-1, IGFBP1, IGFBP2, IGFBP3, IGFBP7, leptin, adiponectin, GDF15, insulin, C-peptide, GIP, GLP-1, and IL-6.

At each time point, the fasting specimen was taken via venipuncture after a minimum of 12 h overnight fast. Once this was collected, the participants were asked to take their morning tablet (metformin or placebo) and then consume a beverage containing 75 grams of glucose. Ninety minutes after glucose consumption, the postprandial serum sample was obtained.

### Blood sample processing

For each serum sample, approximately 6 mL of whole blood were collected and allowed to clot at fridge or room temperature. The sample was then centrifuged between 4°C and room temperature at a low speed to pellet cells, leaving a supernatant of approximately 3 mL of straw-colored serum. Five plastic freezer tubes were used for aliquoting, and 0.3–0.4 mL of serum were placed into each tube. The tubes were marked with the study name, center–subject-anonymized identification, date, time, and number of tubes stored. The serum aliquots were then placed directly into a −80°C freezer until shipping. If a −80°C freezer was not readily accessible, aliquots were allowed to be stored in a −20°C freezer for up to 10 days prior to being transferred to −80°C. Each blood draw’s information was recorded on a sample log. Serum aliquots were shipped annually from each participating institution to a centralized laboratory (https://www.mcgill.ca/assaylab/) for standardized processing. ELISAs were performed on serum samples collected across six study visits from 79 patients, resulting in a total of 474 samples. Each assay was performed in duplicate using commercially available ELISA reagents and carried out according to the manufacturer’s instructions. The ELISAs used in this study are as follows: IGF-1 (Cat. AL-121, Ansh Labs, USA), IGFBP2 (Cat. AL-140, Ansh Labs, USA), IGFBP3 (Cat. AL-120, Ansh Labs, USA), IGFBP7 (Cat. ELH-IGFBPRP1, RayBiotech Life, Inc., USA), leptin (Cat. SLP00, R&D Systems, Inc., USA), adiponectin (Cat. SRP300, R&D Systems, Inc., USA), GDF-15 (Cat. SGD150, R&D Systems, Inc., USA), insulin (Cat. 10-1113-10, Mercodia, Sweden), C-peptide (Cat. AL-151, Ansh Labs, USA), GLP-1 (Cat. 10-1278-01, Mercodia, Sweden), GIP (Cat. 10-1258-01, Mercodia, Sweden), IL-6 (Cat. SS600C, R&D Systems, Inc., USA), and IGFBP1 (Cat. 11-IGFHU-E01, ALPCO, USA).

### Statistical analysis

Descriptive statistics were used to present the study variables. Mean and standard deviations were reported for continuous variables. Frequency and proportions were reported for categorical variables. Two-tailed independent *t*-tests were used to determine whether significant changes in laboratory values were evident in patients receiving metformin vs placebo. The correlation between categorical variables and the study group (metformin and placebo) was conducted using chi-square tests. When comparing two independent proportions, *t*-test of proportions was used. In the placebo group, independent *t*-tests were also conducted to evaluate analyte changes from baseline at 9 and 12 months, in both fasting and postprandial states. Paired *t*-tests were used to evaluate within-subject differences from baseline to 9, 12, 18, and 24 months in the placebo and metformin arms separately. Two-tailed tests and a *P*-value <0.05 were used for statistical significance. SPSS (IBM SPSS Statistics for Windows, version 28, IBM Corp., USA) was used for all statistical analyses.

## Results

Between July 12, 2018, and November 24, 2023, a total of 192 patients were evaluated for eligibility for the PRIME trial. Of them, 166 met the inclusion criteria and were randomized, with 112 assigned to the metformin group and 54 to the placebo group. The baseline patient and treatment characteristics are summarized in Table 1.

**Table 1:** Baseline patient characteristics. Data are mean (SD) or *n* (%) unless specified otherwise.

	Whole group	Metformin group	Placebo group
Age*	70.7 (65.6–75.5)	70.9 (65.7–76.2)	69.2 (64.7–73.6)
Ethnicity			
European	119 (88.1%)	79 (87.8%)	40 (88.9%)
Others	16 (11.9%)	11 (12.2%)	5 (11.1%)
Tumor and treatment characteristics			
PSA, mg/L	20.4 (37.1)	20.4 (31.3)	20.6 (47.3)
ADT duration, months*	17 (9.9–27.5)	18 (10.2–26.4)	17 (9.9–27.9)
Anthropomorphic characteristics			
Weight, kg	89.3 (16.4)	86 (15.8)	95.8 (15.7)
Waist circumference, cm	104.4 (12.2)	102 (11.4)	109.1 (12.5)
BMI, kg/m^2^	29.1 (4.6)	28 (4.5)	31.2 (4)
Systolic BP, mmHg	138.1 (15.5)	138.1 (16.2)	138.2 (14.2)
Diastolic BP, mmHg	79.7 (9)	79.8 (9.2)	79.5 (10.6)
Glucose metabolism			
HbA1c, %	5.6 (0.3)	5.6 (0.3)	5.7 (0.3)
Fasting blood glucose, mmol/L	5.6 (0.5)	5.6 (0.5)	5.6 (0.4)
Lipid metabolism			
Triglycerides, mmol/L	1.3 (0.6)	1.2 (0.5)	1.3 (0.7)
HDL cholesterol, mmol/L	1.4 (0.4)	1.4 (0.4)	1.3 (0.3)
LDL cholesterol, mmol/L	2.8 (1.0)	2.9 (1.0)	2.6 (0.9)
Total cholesterol, mmol/L	4.7 (1.1)	4.8 (1.1)	4.3 (1.1)

* Median (IQR).

ADT, androgen deprivation therapy.

The primary and secondary endpoints of the PRIME trial have been previously reported (14) and are summarized in Table 2. Briefly, the prevalence of MS was not significantly different between the metformin and placebo groups across all time points (Table 2). However, multiple secondary endpoints, including body weight, waist circumference, BMI, and HbA1c, were all significantly improved with metformin compared to placebo (Table 2).

**Table 2:** Key outcome variables from the PRIME trial.

Variable	9 months	12 months	18 months	24 months
Presence of metabolic syndrome				
Placebo	61%	56.80%	67.60%	59.30%
Metformin	48.80%	51.80%	54.80%	43.80%
*P* value	0.2	0.58	0.21	0.2
**Mean (SEM) changes from baseline**				
Weight, kg				
Placebo	+1.83 (3.84)	+1.76 (3.88)	+1.78 (4.68)	+2.04 (4.24)
Metformin	−0.91 (3.97)	−0.33 (3.89)	+0.66 (8.53)	+1.31 (4.29)
*P* value	<0.001	0.004	0.48	0.46
Waist circumference, cm				
Placebo	+2.91 (5.75)	+3.3 (6.03)	+3.85 (6.11)	+3.64 (6.85)
Metformin	+0.84 (4.30)	+1.86 (5.08)	+1.82 (3.81)	+3.18 (4.49)
*P* value	0.03	0.15	0.04	0.71
BMI, kg/m^2^				
Placebo	+0.57 (1.29)	+0.39 (1.55)	+0.56 (1.54)	+0.61 (1.19)
Metformin	−0.35 (1.16)	−0.05 (1.37)	+0.03 (1.34)	+0.35 (1.38)
*P* value	<0.001	0.12	0.09	0.45
HbA1c, %				
Placebo	+0.08 (0.26)	+0.08 (0.27)	+0.11 (0.29)	+0.09 (0.22)
Metformin	−0.02 (0.23)	+0.03 (0.27)	+0.04 (0.54)	+0.09 (0.29)
*P* value	0.02	0.03	0.13	0.89
**Mean (SEM) values at different time points**				
Weight, kg				
Placebo	97.86 (16.18)	97.22 (16.29)	95.48 (17.32)	97.85 (18.26)
Metformin	85.44 (16.38)	86.76 (15.28)	87.76 (19.99)	87.66 (14.87)
*P* value	<0.001	<0.001	0.057	0.007
Waist circumference, cm				
Placebo	111.89 (12.54)	112.12 (12.45)	111.98 (13.05)	113.32 (12.64)
Metformin	102.93 (12.20)	104.54 (11.56)	105.02 (11.81)	106.25 (10.2)
*P* value	<0.001	0.001	0.007	0.007
BMI, kg/m^2^				
Placebo	31.94 (4.33)	31.68 (4.46)	31.78 (4.61)	31.73 (3.81)
Metformin	27.8 (4.70)	28 (3.99)	27.96 (4.04)	28.16 (3.78)
*P* value	<0.001	<0.001	<0.001	0.001
HbA1c, %				
Placebo	5.74 (0.36)	5.75 (0.39)	5.74 (0.36)	5.72 (0.27)
Metformin	5.53 (0.37)	5.52 (0.39)	5.5 (0.63)	5.6 (0.34)
*P* value	0.002	0.003	0.041	0.14

A total of 47 patients from the metformin arm and 32 patients from the placebo arm underwent optional serum collection and were included in the present analysis of additional analytes. Within the placebo group, when comparing baseline fasting and postprandial analyte measures to 9- and 12-month values, leptin, IGFBP3, and GDF15 were found to have increased significantly at all time points (Table 3). Fasting adiponectin also increased from baseline to 9 and 12 months but did not reach significance at 12 months (Table 3). Similarly, fasting and postprandial IGFBP7 increased from baseline across all time points but did not reach significance at 12 months (Table 3). C-peptide showed significant increases from baseline in fasting values at both 9 and 12 months, but not with the postprandial values (Table 3). Fasting GIP demonstrated an increasing trend at 9 months, and the improvement was significant at 12 months (Table 3). Although numerical increases were identified in fasting and postprandial IL-6 values at 9 and 12 months compared to baseline, the changes did not reach statistical significance (Table 3). Fasting insulin demonstrated a similar trend with a non-significant increase at 9 and 12 months compared with baseline (Table 3). The remaining analytes demonstrated minor changes from baseline that did not reach statistical significance (Table 3).

**Table 3:** Mean (SD) changes in metabolite values from baseline in the placebo group, fasting (F) and postprandial (PP). ELISAs were performed on serum samples collected across six study visits from 79 patients, resulting in a total of 474 samples.

Variable	9 months F	12 months F	9 months PP	12 months PP
IGF-1, ng/mL	−18.59 (97.93)	−2.94 (90.85)	−4.35 (76.43)	+4.47 (81.95)
*P* value	0.29	0.86	0.75	0.76
IGFBP1, ng/m	−0.21 (1.23)	+0.17 (1.71)	−0.20 (1.42)	+0.08 (1.71)
*P* value	0.34	0.57	0.42	0.78
IGFBP2, ng/mL	−28.98 (132.75)	−9.81 (183.96)	−37.44 (137.53)	−18.53 (181.19)
*P* value	0.23	0.77	0.13	0.57
IGFBP3, ng/mL	+793.49 (814.67)	+622.39 (763.33)	+742.77 (936.70)	+648.23 (939.27)
*P* value	4.98E−06	6.50E−05	9.32E−05	4.77E−04
IGFBP7, ng/mL	+10.71 (22.84)	+26.36 (96.19)	+12.09 (32.08)	+22.1 (89.54)
*P* value	0.01	0.13	0.04	0.17
Leptin, pg/m	+9,629.56 (10,163.13)	+10,390.72 (12,625.06)	+9,990.28 (9,562.57)	+9,959.69 (11,028.08)
*P* value	7.65E−06	5.75E−05	1.59E−06	1.57E−05
Adiponectin, ng/mL	+1,685.00 (3,320.27)	+682.19 (2,132.68)	+1,671.94 (3,223.80)	+761.41 (1,944.18)
*P* value	0.01	0.08	0.01	0.03
GDF15, pg/mL	+196.66 (383.81)	+246.16 (517.53)	+177.75 (416.49)	+226.69 (503.83)
*P* value	0.01	0.01	0.02	0.02
Insulin, mU/L	+6.52 (18.66)	+1.99 (7.82)	−4.90 (52.7)	−17.25 (66.00)
*P* value	0.06	0.16	0.6	0.15
C-peptide, ng/mL	+0.87 (1.72)	+0.44 (1.20)	+0.11 (2.12)	+0.12 (2.23)
*P* value	0.01	0.05	0.76	0.76
GLP-1, pM	+0.10 (3.28)	+0.16 (3.86)	−2.71 (12.25)	−3.22 (11.96)
*P* value	0.87	0.81	0.22	0.15
GIP, pM	+4.02 (14.79)	+1.75 (4.52)	+0.99 (16.69)	−1.79 (13.16)
*P* value	0.15	0.04	0.74	0.45
IL-6, pg/mL	+2.07 (7.86)	+0.96 (4.30)	+1.82 (6.80)	+0.79 (3.56)
*P* value	0.15	0.22	0.14	0.22

**Figure 1:**
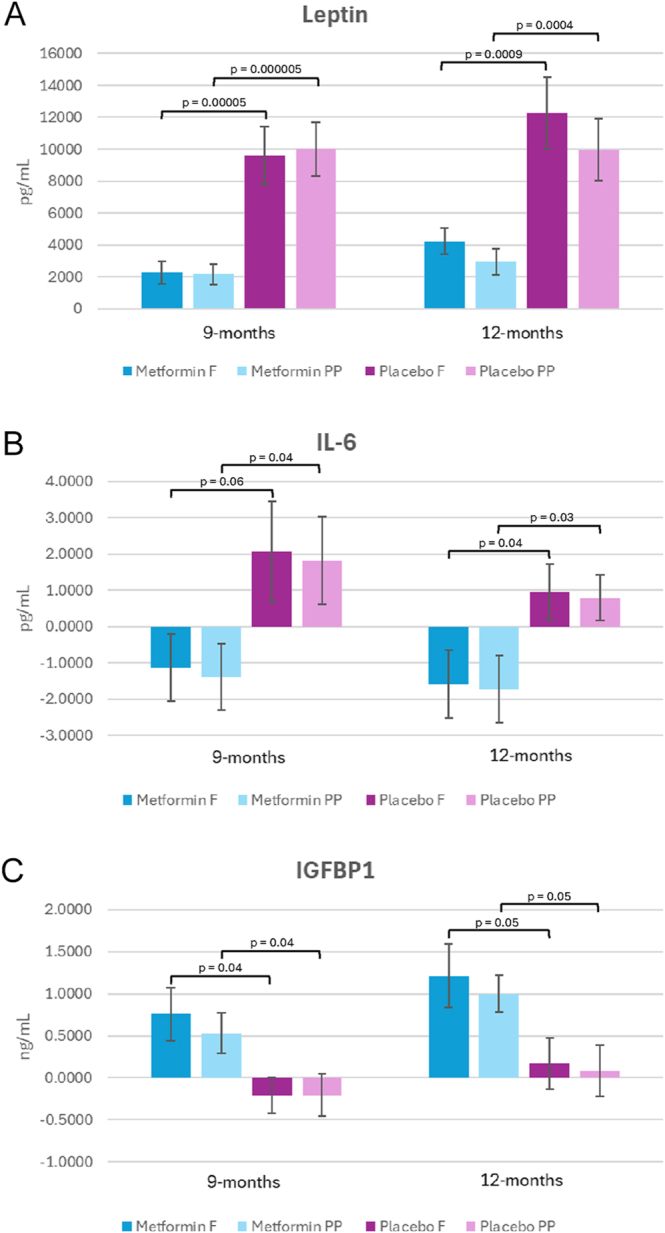
Mean changes in leptin (A), IL-6 (B), and IGFBP1 (C) from baseline in the metformin vs placebo groups. In PCa patients treated with ADT, commercially available ELISAs were performed on serum samples collected across six study visits from 79 patients, resulting in a total of 474 samples. The assays used are as follows for each analyte: leptin (Cat. SLP00, R&D Systems, Inc., USA), IL-6 (Cat. SS600C, R&D Systems, Inc., USA), and IGFBP1 (Cat. 11 -IGFHU -E01, ALPCO, USA). Two-tailed independent t-tests were used to determine whether significant changes in laboratory values were evident in patients receiving metformin vs placebo at 9 and 12 months. Both fasting (F) and postprandial (PP) values were assessed at these time points. The error bars represent the standard error of the mean.

Comparing the metformin group to the placebo group, the mean leptin values increased markedly in the placebo group but significantly less in the metformin group across all time points (Fig. 1A, Tables 4 and 5). The mean fasting IL-6 values decreased with metformin compared with placebo at all time points but did not reach statistical significance at 9 months (Fig. 1B, Tables 4 and 5). The mean IGFBP1 values increased more with metformin compared with placebo at all time points (Fig. 1C, Tables 4 and 5).

**Table 4:** Mean (SD) changes in fasting outcome variables from baseline. ELISAs were performed on serum samples collected across six study visits from 79 patients, resulting in a total of 474 samples.

Variable	9 months	12 months
IGF-1, ng/mL		
Placebo	−18.59 (97.93)	−2.94 (90.85)
Metformin	−15.80 (75.90)	−6.18 (74.39)
*P* value	0.892	0.87
IGFBP1, ng/mL		
Placebo	−0.21 (1.23)	+0.17 (1.71)
Metformin	+0.76 (2.16)	+1.21 (2.60)
*P* value	0.03	0.05
IGFBP2, ng/mL		
Placebo	−28.98 (132.75)	−9.81 (183.96)
Metformin	+13.49 (137.10)	+19.56 (145.43)
*P* value	0.17	0.45
IGFBP3, ng/mL		
Placebo	+793.49 (814.67)	+622.39 (763.33)
Metformin	+597.16 (598.24)	+553.09 (596.19)
*P* value	0.25	0.67
IGFBP7, ng/mL		
Placebo	+10.71 (22.84)	+26.36 (96.19)
Metformin	+11.12 (44.91)	+19.41 (48.48)
*P* value	0.96	0.71
Leptin, pg/mL		
Placebo	+9,629.56 (10,163.13)	+10,390.72 (12,625.06)
Metformin	+2,284.28 (4,843.06)	+3,164.36 (5,709.88)
*P* value	4.98E−05	9.20E−04
Adiponectin, ng/mL		
Placebo	+1,685.00 (3,320.27)	+682.19 (2,132.68)
Metformin	+1,633.49 (1,987.13)	+1,259.72 (2,041.56)
*P* value	0.94	0.23
GDF15, pg/mL		
Placebo	+196.66 (383.81)	+246.15 (517.53)
Metformin	−94.13 (4,594.85)	−33.72 (4,491.79)
*P* value	0.67	0.67
Insulin, mU/L		
Placebo	+6.52 (18.66)	+1.99 (7.82)
Metformin	+0.01 (3.30)	+0.84 (6.69)
*P* value	0.07	0.5
C-peptide, ng/mL		
Placebo	+0.87 (1.72)	+0.44 (1.20)
Metformin	+0.00 (1.27)	+0.48 (1.85)
*P* value	0.02	0.91
GLP-1, pM		
Placebo	+0.10 (3.28)	+0.16 (3.86)
Metformin	+2.13 (3.83)	+2.18 (3.22)
*P* value	0.01	0.02
GIP, pM		
Placebo	+4.02 (14.79)	+1.75 (4.52)
Metformin	+0.98 (6.75)	+1.81 (5.40)
*P* value	0.3	0.96
IL-6, pg/mL		
Placebo	+2.07 (7.86)	+0.96 (4.30)
Metformin	−1.14 (6.36)	−1.58 (6.37)
*P* value	0.06	0.04

**Table 5:** Mean (SD) changes in postprandial outcome variables from baseline. ELISAs were performed on serum samples collected across six study visits from 79 patients, resulting in a total of 474 samples.

Variable	9 months	12 months
IGF-1, ng/mL		
Placebo	−4.35 (76.43)	+4.47 (81.95)
Metformin	−12.64 (77.42)	−1.15 (78.90)
*P* value	0.64	0.76
IGFBP1, ng/mL		
Placebo	−0.20 (1.42)	+0.08 (1.71)
Metformin	+0.53 (1.65)	+1.00 (2.28)
*P* value	0.04	0.05
IGFBP2, ng/mL		
Placebo	−37.44 (137.53)	−18.53 (181.18)
Metformin	+9.85 (138.32)	+10.56 (131.62)
*P* value	0.14	0.44
IGFBP3, ng/mL		
Placebo	+742.77 (936.70)	+648.23 (939.28)
Metformin	+607.74 (541.91)	+627.02 (583.42)
*P* value	0.47	0.91
IGFBP7, ng/mL		
Placebo	+12.09 (32.08)	+22.10 (89.54)
Metformin	+7.49 (47.73)	+15.73 (44.95)
*P* value	0.61	0.71
Leptin, pg/mL		
Placebo	+9,990.28 (9,562.57)	+9,959.69 (11,028.08)
Metformin	+2,153.09 (4,494.03)	+2,952.23 (5,685.77)
*P* value	5.37E−06	4.02E−04
Adiponectin, ng/mL		
Placebo	+1,671.94 (3,223.80)	+761.41 (1,944.18)
Metformin	+1,583.06 (2,063.38)	+1,424.21 (1,994.18)
*P* value	0.89	0.15
GDF15, pg/mL		
Placebo	+177.75 (416.49)	+226.69 (503.83)
Metformin	−131.11 (4,252.63)	−64.91 (4,197.69)
*P* value	0.62	0.64
Insulin, mU/L		
Placebo	−4.90 (52.7)	−17.25 (66.00)
Metformin	−4.00 (53.51)	−0.72 (41.43)
*P* value	0.94	0.21
C-peptide, ng/mL		
Placebo	+0.11 (2.12)	+0.12 (2.23)
Metformin	−0.43 (2.49)	−0.05 (1.87)
*P* value	0.3	0.73
GLP-1, pM		
Placebo	−2.71 (12.25)	−3.22 (11.96)
Metformin	+1.60 (7.78)	+0.55 (7.34)
*P* value	0.09	0.12
GIP, pM		
Placebo	+0.99 (16.69)	−1.79 (13.16)
Metformin	+2.65 (13.69)	+3.37 (21.08)
*P* value	0.64	0.96
IL-6, pg/mL		
Placebo	+1.82 (6.80)	+0.79 (3.56)
Metformin	−1.39 (6.30)	−1.73 (6.30)
*P* value	0.04	0.03

Fasting GLP-1 increased significantly with metformin compared with placebo at both 9 and 12 months (Tables 4 and 5). Improvements were noted in postprandial values as well but did not reach statistical significance (Tables 4 and 5). Fasting C-peptide increased significantly in the placebo arm compared with the metformin arm only at 9 months (Tables 4 and 5).

Some favorable changes in mean values were observed at various time points for insulin, adiponectin, IGFBP2, IGFBP3, and GDF-15 (Tables 4 and 5). However, none of these were statistically significant. IGF-1 and IGFBP7 showed no significant changes with metformin compared with placebo (Tables 4 and 5).

The cumulative incidence of toxicity was low, with no significant difference between arms (7 vs 3 events; *P* = 0.9). There was only one patient with grade 2 toxicity. No patients in either arm experienced severe toxicities (grade ≥ 3), and no participants withdrew from the study due to metformin toxicity.

## Discussion

In this analysis of serum specimens from the PRIME trial, we evaluated laboratory analytes associated with MS and T2DM and hypothesized that ADT would cause adverse changes that could be mitigated by metformin.

Leptin proved to be a noteworthy analyte in our study. Increases in this analyte after the initiation of ADT were highly significant, and metformin significantly attenuated this increase. Our findings extend the results of a cross-sectional study reported by Basaria *et al.*, in which higher leptin levels were observed in men after ADT compared with subjects not on ADT, but this study did not report the time course of changes in individual patients nor metformin effects (5). Elevated leptin levels have been shown to correlate with obesity and MS as demonstrated in a recent meta-analysis reported by Hernández-Díaz *et al.* (20). In addition, Wu *et al.* indicated a significant elevation in leptin in patients with diabetes compared with controls (32). Therefore, the attenuation of the increase in leptin by metformin may reduce metabolic toxicity of androgen deprivation in this patient population.

Several studies have shown an association between aggressive PCa and elevated leptin levels (33, 34, 35). In a recent meta-analysis, Burton *et al.* showed a weak positive correlation between increased leptin levels and aggressive PCa. They acknowledged that the available data were suboptimal (36), so the association between leptin and prostate cancer outcomes may have been over- or underestimated. Preclinical data have shown that leptin antagonism inhibits prostate cancer xenograft growth and progression (37). In addition, Li *et al.* assessed leptin levels in patients with metastatic castration-resistant prostate cancer receiving docetaxel. They demonstrated that patients with high leptin levels had shorter progression-free survival and shorter overall survival compared to men with low leptin levels (38). Recently, the STAMPEDE trial has published results assessing the impact of metformin treatment on overall survival in patients with metastatic PCa treated with ADT. In a prespecified subgroup analysis, they demonstrated that patients with high-volume metastatic disease derived an overall survival benefit from metformin treatment. This subgroup analysis was not powered, and the authors indicated that these results should be interpreted as hypothesis generating. Leptin was not assessed in this study (39). However, given the association between elevated leptin levels and more aggressive forms of prostate cancer (33, 34, 35), our findings align with those of the STAMPEDE trial, where the attenuation of leptin we have demonstrated may induce the oncologic benefits in patients with high-volume metastatic disease. Metformin treatment was also tested in PCa patients with low-risk disease on active surveillance in a randomized controlled trial by Fleshner *et al.* No statistically significant difference in progression-free survival was observed between patients treated with metformin compared with placebo (40). This also aligns with our findings in that the benefit of metformin treatment may be derived in more aggressive forms of PCa. Longer-term studies are needed in patients with high-grade, aggressive prostate cancer or high-volume metastatic disease to determine whether the addition of metformin to ADT would influence prostate cancer outcomes and/or leptin-related adverse effects.

Other notable analytes in this study were IGFBP1 and IL-6. Decreased IGFBP1 and elevated IL-6 levels are associated with impaired glucose tolerance, impaired fasting glucose, and an increased risk of MS and T2DM (16, 17, 29). IL-6 is also associated with several complications of T2DM, including diabetic kidney disease, diabetic peripheral neuropathy, and cognitive impairment (31). Our analysis demonstrates that patients on metformin had increased levels of IGFBP1 across all time points compared with controls. There was a notable decrease in IL-6 levels in the metformin group compared with placebo. The decrease was significant across all time points except for the fasting level at 9 months. These findings indicate the potential for metformin to decrease the occurrence of T2DM and the resultant complications in patients treated with ADT.

GLP-1 is an incretin hormone, which plays a key role in the pathophysiology of T2DM and functions to regulate blood glucose levels through augmenting insulin secretion. This function is abnormal in T2DM, and GLP-1 receptor agonists (GLP-1RA) normalize blood glucose in this population (41). Preclinical studies have shown that GLP-1RA attenuate prostate cancer growth in cell lines exhibiting GLP-1 receptor expression (42, 43). While several mechanisms have been hypothesized for the anti-tumoral effect, the primary driver is suspected of being due to the inhibition of the ERK–MAPK pathway, which is a main signaling pathway in prostate cancer cell proliferation (42). A cohort study completed by Skriver *et al.* found an inverse association with PCa risk in patients treated with GLP-1RA compared with those treated with basal insulin, leading to the conclusion that GLP-1RA may protect against prostate cancer (44). Similar to metformin, the use of GLP-1RA may offer oncologic benefits, but this requires further exploration as no prospective clinical studies have evaluated GLP-1RA as a therapeutic intervention for prostate cancer. The PRIME trial cohort did not include any users of GLP-1RA. Our findings show an increase in GLP-1 levels in patients treated with metformin compared with placebo, which was significant in fasting values but did not reach significance in postprandial values. The improvement in GLP-1 levels in our study is further evidence of a potential oncologic benefit of adding metformin to ADT.

Elevated IGFBP3 and GDF15 levels have been associated with an increased incidence of T2DM and MS (15, 45). We noted significant increases in IGFBP3 and GDF15 from baseline in the placebo arm. Our results concur with the secondary analysis of the CHAARTED trial, which showed IGFBP3 elevation in patients with metastatic PCa treated with ADT (46). However, these changes were not improved in the metformin arm.

Previous studies have linked low adiponectin levels to T2DM and MS (47, 48). In our study, adiponectin increased in patients receiving ADT and this increase was significant across all time points except at 12 months in the fasting state. This paradoxical increase in adiponectin is likely due to an inverse relationship between androgen levels and adiponectin (49). A prospective study in healthy men demonstrated that serum adiponectin levels significantly increased following androgen suppression with a GnRH antagonist. In addition, adiponectin levels decreased when participants were administered supraphysiologic doses of testosterone (50). Therefore, the rise in adiponectin seen in our study may be explained by androgen suppression having a direct or an indirect effect on circulating adiponectin levels. Further research is required to determine the impact of adiponectin rise in the context of ADT.

The other analytes evaluated in our study showed significant but inconsistent changes, or no significant changes at follow-up. Therefore, meaningful conclusions could not be drawn.

The strengths of the PRIME trial include its double-blinded, multicenter phase III design, standardized sample handling, and centralized assays and data analysis. This analysis shares the same limitations as the PRIME study, which was terminated early due to drug supply limitations, resulting in a smaller-than-intended sample size. A baseline imbalance between the two arms was possible as participants were not stratified for the presence of MS. Diet, exercise, and steroid supplementation were not controlled for in this study, which could have had an impact on the analyte levels. For the correlative analysis specifically, this was an opt-in laboratory assessment among participants. Therefore, this analysis was limited to a smaller sample size compared with the primary study (ELISAs were performed on serum samples collected across six study visits from 79 patients, resulting in a total of 474 samples), limiting the overall statistical power.

This correlative analysis of a phase III randomized controlled trial shows the favorable effects of metformin on the biochemical profile of PCa patients treated with ADT. Most notably, the substantial increase in leptin observed in patients treated with ADT was significantly subdued with metformin. Our results also indicate the numerous adverse metabolic changes in the ADT-alone group associated with an increased risk of MS and T2DM. Metformin is a simple and safe strategy to mitigate this risk and perhaps improve the metabolic profile and outcomes associated with aggressive forms of prostate cancer. While we conclude that combining metformin with ADT in prostate cancer treatment is safe and reduces unwanted effects on biomarkers associated with MS, longer-term studies are required to determine whether this combination will favorably impact prostate cancer outcomes and/or attenuate the metabolic toxicities of ADT.

## Declaration of interest

Drs Eigl, Ghosh, Kim, Brundage, Pollak, Courneya, and Usmani report peer-reviewed competitive grant funding from Prostate Cancer Canada. Dr Bouchard reports support from TerSera, AstraZeneca, and Astellas, outside of the submitted work. Dr Courneya reports travel grants and consulting fees from Amgen, Astellas, AstraZeneca, Bristol Myers Squibb, Janssen, Novartis, Merck, Point Biopharma, Pfizer, and Roche pharmaceuticals, outside of the submitted work.

## Funding

The PRIME trial was supported by the Movember Translation Grant by Prostate Cancer Canada (Grant No. 400363).
